# Loop ostomy following laparoscopic low anterior resection for rectal cancer after neoadjuvant chemoradiotherapy

**DOI:** 10.1186/s40001-018-0325-x

**Published:** 2018-05-22

**Authors:** Xin Wu, Guole Lin, Huizhong Qiu, Yi Xiao, Bin Wu, Miner Zhong

**Affiliations:** Department of General Surgery, Peking Union Medical College Hospital, Chinese Academy of Medical Sciences and Peking Union Medical College, No. 1 Shuaifuyuan, Wangfujing, Dongcheng District, Beijing, 100730 China

**Keywords:** Temporary loop ostomy, Loop ileostomy, Loop transverse colostomy, Rectal cancer, Neoadjuvant chemoradiotherapy

## Abstract

**Background:**

Both loop ileostomy (LI) and loop transverse colostomy (LTC) could achieve absolute fecal diversion and have several advantages. This study compared LI and LTC following laparoscopic low anterior resection for rectal cancer after neoadjuvant chemoradiotherapy.

**Methods:**

Between January 2009 and December 2016, 186 patients who underwent laparoscopic low anterior resection for rectal cancer and loop ostomy were included. All patients received preoperative neoadjuvant chemoradiotherapy. Of these, 77 underwent LI and 109 underwent LTC. Demographic characteristics, operative details, and complications were analyzed.

**Results:**

In the fecal diversion period, the LTC group showed significantly less dermatitis (*p* = 0.001) and electrolyte disturbance (*p* = 0.002), while LI group showed significantly shorter time to first defecation (*p* = 0.006) and lower incidence of parastomal hernia (*p* = 0.014). In the stoma closure period, a significantly higher incidence of wound infection was found in LTC group (*p* = 0.001).

**Conclusions:**

Both LI and LTC have advantages and disadvantages. For its lower wound infection rate, lower incidence of parastomal hernia, and shorter time to first defecation, LI is recommended for all patients except those with potential electrolyte disturbance and sensitive skin.

## Background

The development of laparoscopic technique and neoadjuvant chemoradiotherapy helps surgeons to challenge the limited capacity for anus preservation after operations for low rectal cancer. The incidence of anastomotic leakage is particularly high if the anastomosis involves the anal canal or distal rectum. Though it remains controversial, ostomy is effective in preventing abdominal contamination, septic shock, and other complications in case of anastomotic leakage [1–4]. This would be more pronounced for patients who have finished neoadjuvant chemoradiotherapy.

Both loop ileostomy (LI) and loop transverse colostomy (LTC) could achieve absolute fecal diversion and reduce the incidence of anastomotic leakage. In the literature published in English, some favor LI, whereas others prefer LTC [5–8]. To the best of our knowledge, no dedicated study about ostomy following laparoscopic low anterior resection for rectal cancer after neoadjuvant chemoradiotherapy has been performed. Therefore, we designed a retrospective clinical study, collected the data in our center, and compared LI and LTC in patients with the same background.

## Methods

### Patients

Between January 2009 and December 2016, a total of 1298 patients underwent laparoscopic anterior resection for rectal cancer in two surgical teams of our center. Routine fecal diversion was performed if neoadjuvant chemoradiotherapy had been finished before surgery. LI was the standard procedure in team A and LTC was the preferred method in team B. All the medical records were reviewed systematically and carefully. Inclusion criteria included: (i) neoadjuvant chemoradiotherapy was indicated and finished before surgery; (ii) patients received follow-up and underwent stoma closure at our center. Patients with multiple gastrointestinal cancers or inflammatory bowel disease were excluded. Finally, 77 patients who underwent LI and 109 patients who underwent LTC were selected. All the patients were classified as clinical stages II or III before treatment.

### Treatment

Preoperative chemotherapy contained two cycles of 5-fluorouracil and leucovorin for 5 days in the 1st and the 5th weeks. Concurrent radiation therapy comprised 45 Gy delivered to the pelvis and 5.4 Gy boost to the primary tumor over a period of 5 weeks. Surgery was performed 8–12 weeks after neoadjuvant chemoradiotherapy. Another four cycles of chemotherapy contained 5-fluorouracil and leucovorin were recommended after surgery.

Loop ostomy was performed after laparoscopic low anterior resection. The stoma was located in the lower abdomen for LI, and in the upper abdomen for LTC. The loop was opened on postoperative day 2 to protect the incision. Stoma closure was performed after 6 months follow-up at least. Hand-sewn suture with end to end anastomosis or closure of the anterior wall was the standard procedure. Oral laxative was given to patients the day before surgery for bowel preparation. Prophylactic antibiotics were administered through a peripheral vein before anesthesia induction.

### Data collection

Clinical data were collected from both inpatient and outpatient medical records. Demographic characteristics, operative details, and complications were analyzed. Complications were studied in the fecal diversion and stoma closure periods. The former included dermatitis, renal insufficiency, parastomal hernia, stoma prolapse, retraction, necrosis, and electrolyte disturbance; whereas the latter included wound infection, anastomotic leakage, stenosis, and incisional hernia.

Dermatitis was defined as skin symptoms around stoma that lasted more than 1 week and need professional colostomy care in hospitals. Renal insufficiency was determined by a raise in creatinine beyond 104 μmol/L. Electrolyte disturbance was defined as hyperkalemia (a raise of potassium beyond 5.5 mmol/L), hypokalemia (a decrease of potassium below 3.5 mmol/L), hyponatremia (a decrease of sodium below 135 mmol/L), and hypocalcemia (a decrease of total calcium below 2.1 mmol/L) that lasted more than 3 days and required medication. Wound infection was defined as the situation that need original suture removing and regular wound dressing.

### Statistical analysis

Statistical analysis was carried out by an independent statistician. The Statistical Package for Social Sciences software (SPSS, version 19.0, Chicago, IL, USA) was used. Differences between study groups were analyzed by *χ*^2^ test, Fisher’s exact test, and Student’s *t* test as appropriate. A *p* value < 0.05 was considered to statistically significant. All data were represented as mean ± standard deviation or median as appropriate.

## Results

A total of 186 patients were divided into two groups: LI group with 77 patients, and LTC group with 109 patients. Males (*n* = 123) comprised 66.1% of the patients, and 33.9% were females (*n* = 63). Mean age of the 186 patients was 59.5 ± 11.8 years (range 29–85 years). Mean body mass index (BMI) was 24.0 ± 2.4 kg/m^2^. Sex distribution, mean ages and mean BMI in both groups were similar (*p* > 0.05). Patients with coronary disease (*n* = 72) comprised 38.7%, those with diabetes (*n* = 51) 27.4%, those with COPD (chronic obstructive pulmonary disease) (*n* = 14) 7.5%, those with cerebral infarction (*n* = 10) 5.4%, and those with renal insufficiency (*n* = 8) 4.3%. Distribution among both groups is shown in Table 1.

**Table 1:** Demographic data of LI and LTC groups

	Total (*n* = 186)	LI (*n* = 77)	LTC (*n* = 109)	*p* value
Gender				
Males	123	52	71	0.734
Females	63	25	38	
Age in years	59.5 ± 11.8	57.9 ± 10.2	60.7 ± 12.7	0.091
BMI (kg/m^2^)	24.0 ± 2.4	23.7 ± 2.7	24.2 ± 2.2	0.186
Coronary disease	72	31	41	0.715
Diabetes	51	21	30	0.970
COPD	14	5	9	0.653
Cerebral infarction	10	3	7	0.673
Renal insufficiency	8	4	4	0.890

*LI* loop ileostomy, *LTC* loop transverse colostomy, *BMI* body mass index, *COPD* chronic obstructive pulmonary disease

### Fecal diversion

Mean hospital stay was 13.4 ± 4.7 days (range 8–35 days) in LI group, and 14.3 ± 4.8 days (range 8–34 days) in LTC group. Time to first defecation was after 1.5 ± 1.0 days in LI group and after 2.2 ± 2.0 days in LTC group (*p* = 0.006). For the complication rates, LTC group showed significantly less dermatitis (*p* = 0.001) and electrolyte disturbance (*p* = 0.002), while LI group showed significantly lower incidence of parastomal hernia (*p* = 0.014). Other parameters did not show any statistical difference between both groups (*p* > 0.05). The detailed data and complication rates are shown in Table 2.

**Table 2:** Data and complication rates during the fecal diversion period

	Total (*n* = 186)	LI (*n* = 77)	LTC (*n* = 109)	*p* value
Hospital stay (days)	13.9 ± 4.7	13.4 ± 4.7	14.3 ± 4.8	0.210
Time to first defecation (days)	1.9 ± 1.7	1.5 ± 1.0	2.2 ± 2.0	0.006
Dermatitis	20	15	5	0.001
Renal insufficiency	14	8	6	0.214
Parastomal hernia	29	6	23	0.014
Stoma prolapse	4	1	3	0.873
Retraction	1	0	1	1.000
Necrosis	1	0	1	1.000
Electrolyte disturbance	26	18	8	0.002

*LI* loop ileostomy, *LTC* loop transverse colostomy

### Stoma closure

Mean time during stoma placement and reversal, mean hospital stay, time to first defecation and mean time of the closure operation were analyzed. The differences between both groups were not statistically significant (*p* > 0.05). The median follow-up time after stoma closure was 27 months (range 6–83 months). A significantly higher incidence of wound infection was found in the LTC group than in the LI group (*p* = 0.001). The other complication rates were similar between both groups (*p* > 0.05). Distribution and statistical differences are shown in Table 3.

**Table 3:** Data and complication rates during the stoma closure period

	Total (*n* = 186)	LI (*n* = 77)	LTC (*n* = 109)	*p* value
Days until stoma reversal	204.3 ± 26.9	201.6 ± 31.0	206.2 ± 23.6	0.256
Hospital stay (days)	9.5 ± 3.3	9.1 ± 2.8	9.9 ± 3.5	0.083
Time of closure operation (minutes)	84.1 ± 22.2	81.5 ± 23.2	86.0 ± 21.3	0.172
Time to first defecation (days)	3.5 ± 1.3	3.3 ± 1.0	3.6 ± 1.5	0.136
Wound infection	25	3	22	0.001
Anastomotic leakage	2	1	1	1.000
Stenosis	2	0	2	0.512
Incisional hernia	6	2	4	1.000

*LI* loop ileostomy, *LTC* loop transverse colostomy

## Discussion

Anastomotic leakage is a serious complication after anterior resection for rectal cancer, especially for the patients with low colorectal or coloanal anastomosis. It is associated with additional medical cost, prolonged hospital stay and an increase in mortality of patients with rectal cancer [9, 10]. Though loop stoma for fecal diversion does not abolish the risk of anastomotic leakage and could influence patients’ life tremendously [11], it could decrease the severity of complications when anastomotic leakage occurs [2, 12]. LI and LTC are two major kinds of ostomy. Many studies have been carried out in the past several decades to analyze the better technique [5, 13–15]. Different studies led to different conclusions because both techniques have several advantages. LI is easy to manage and produces less feculent odor [5, 13]. LTC has less complication, such as intestinal obstruction and ileus [16].

The present study only admitted patients who underwent laparoscopic low anterior resection for rectal cancer after neoadjuvant chemoradiotherapy. The patients were divided into two groups according to the different ostomy techniques. Gender and age distribution, BMI, and preexisting morbidities were similar in both groups. The complication rates were analyzed independently for the fecal diversion and stoma closure periods.

During the fecal diversion period, hospital stay was similar in both groups. Time to first defecation was significantly shorter in the LI group (*p* = 0.006). That was also observed in a previous study [5]. Bowel movement recovered more quickly in ileum than in colon because of abundant blood supply and liquid intestinal content. Dermatitis and electrolyte disturbance occurred significantly more in the LI group (*p* < 0.05). Both complications might be explained by the high flow capacity of small bowel feces. If the ileostomy output remains high, more serious complications such as dehydration and renal impairment would be expected [17–21]. Parastomal hernia was more likely to be seen in the LTC group (*p* = 0.014). This might be caused by the larger fascial defect and heavier intestinal contents. The frequency of herniation was a remarkable drawback of LTC, some researchers even recommended LI to avoid this [22]. Stomal prolapse had been proved to be one of the most common complications after ostomy [23]. It occurred in about 2–22% of patients in one report [24]. In the present study, prolapse was only found in four patients. This might be attributed to the good fixation and appropriate length of loops.

During the stoma closure period, days until stoma reversal, hospital stay, duration of closure operation and time to first defecation showed no significant difference between both groups (*p* > 0.05). Bowel imaging before reversal, short ambulation time after surgery, and reasonable diet guide were possible reasons for the similar hospital stay and time to first defecation between both groups. There was a higher incidence of wound infection in the LTC group (*p* = 0.001). That might be attributed to more contaminated intestinal environment of transverse colon. Besides local wound infection, the incidence of systemic infection, such as sepsis, had been proved to be significantly reduced after ileostomy [25].

Though several patients suffered from severe complications such as necrosis and anastomotic leakage, the total rate of all complications was low. This shows that both stoma placement and closure are safe procedures. Most ostomates have physical, social, and psychological problems. Some of them have to accept low quality daily life [26]. The suitable stoma style should be chosen for each patient. According to our experience, LI and LTC had both advantages and disadvantages. LI had significantly high incidence of dermatitis, and electrolyte disturbance. Whereas, LTC had higher wound infection rate, higher incidence of parastomal hernia, and longer time to first defecation.

Though the present study compared LI and LTC in patients with the same background, it was a retrospective study. Patient-volume, registration information, and inspection items could not be designed beforehand. The comparison of life quality between two groups was lack of quantifiable index. Prospective, randomized, controlled and multi-center clinical trials are required for more supporting evidence, with greater reliability and persuasion.

## Conclusions

Both stoma placement and closure are safe procedures. For its lower wound infection rate, lower incidence of parastomal hernia, and shorter time to first defecation, LI is recommended for all patients except those with potential electrolyte disturbance and sensitive skin.

## Abbreviations

LI: loop ileostomy
LTC: loop transverse colostomy
BMI: body mass index
COPD: chronic obstructive pulmonary disease
